# The Main Medicinal Plants in Arid Regions of Uzbekistan and Their Traditional Use in Folk Medicine

**DOI:** 10.3390/plants12162950

**Published:** 2023-08-15

**Authors:** Ozodbek S. Abduraimov, Wenjun Li, Habibullo F. Shomurodov, Ying Feng

**Affiliations:** 1 State Key Laboratory of Desert and Oasis Ecology, Key Laboratory of Ecological Safety and Sustainable Development in Arid Lands, Xinjiang Institute of Ecology and Geography, Chinese Academy of Sciences, Urumqi 830011, China; 2 Institute of Botany, Academy Science Republic of Uzbekistan, Tashkent 100126, Uzbekistan; 3 The Specimen Museum of Xinjiang Institute of Ecology and Geography, Chinese Academy of Sciences, Urumqi 830011, China

**Keywords:** medicinal plants, biodiversity, traditional use, arid zones, Uzbekistan

## Abstract

Seventy percent of the territory of Uzbekistan consists of arid regions. This situation is considered very favorable for plants adapted to a desert climate. Medicinal plants distributed in the arid regions of Uzbekistan have not been studied much. Medicinal plants are considered inexpensive, yet are vital for the lives of local residents. They play a very important role in the traditional healing of ailments. To determine the current state of medicinal plants and enhance their subsequent protection and sustainable use, it is necessary to obtain annual information on the state of their distribution, their population size, and the impact of negative factors on their populations. Based on our field studies, which were conducted during the period from 2012 to 2022 in the arid regions of Uzbekistan, we updated the checklists of the main medicinal plants used in these regions. A total of 529 medicinal species belonging to 70 families and 269 genera were identified in the study region. Several species, including *Peganum harmala* L., *Capparis spinosa* L., *Ferula foetida* (Bunge) Regel, *Glycyrrhiza glabra* L., *Alhagi pseudalhagi* (M. Bieb.) Desv. ex Wangerin, *Lagochilus inebrians* Bunge, *Xanthium strumarium* L., *Silybum marianum* (L.) Gaertn., *Onopordum acanthium* L., *Ziziphora tenuior* L., and *Cichorium intybus* L., are spread over large areas and have been used regularly by the locals since ancient times. These species are common in saline and degraded soils in arid regions of Uzbekistan. Semi-structured interviews were conducted with tabibs (traditional doctors), elders, herders, and residents with experience in traditional healing using medicinal plants. The medicinal value of most plants was based on the interviews with representatives of the local population, which were useful for understanding traditional healing skills and customer service skills.

## 1. Introduction

Uzbekistan is distinguished by its richness of medicinal plants. Native flora species have been used there by humans for many years. Uzbekistan is located in the territory of Central Asia, with the main part consisting of steppes and arid regions. These lands do not have a high annual rainfall [1,2]. The population of Uzbekistan is the highest in Central Asia, with 8.5–9.0 million people living in the arid regions alone [2].

In recent years, much research has been carried out in South Africa on the study of medicinal plants and their importance [3]. Much attention has also been paid to this in other countries worldwide [4,5]. This, in turn, is due to the increased demand for medicinal plants [6].

The traditional knowledge of the local population is considered very important in this regard. Through this knowledge, the population makes extensive use of medicinal plants [7,8]. Effective scientific research has also been carried out on this topic [9]. Most medicinal plants in Uzbekistan, including *Glycyrrhiza glabra* L., are found among the flora of other countries. Much research has also been conducted on the medicinal plants of other countries [10], including those in China [11] and Arab countries [12].

Botanical studies in recent years show that the study of medicinal plants is considered very important. Many people use medicinal plants regularly [13,14], and medicinal plants play an important role in human life [15]. In particular, their use for treating various diseases, as well as the study of their impact on the human body, are some of the most important areas of research [16,17]. Much work has also been conducted around the world on the chemical composition of medicinal plants and their traditional and modern uses [18]. Ethnobotanical research, as well as the correct use of medicinal plants, is considered important in this because these processes are associated with improving human life [19]. It is considered important to correctly analyze medicinal plants and recommend them to humans because plants can undergo some changes in different conditions, which also affect their composition [20].

The history and future of medicinal plants are considered important research areas because these plants have been used by humans for several centuries. The older literature is also important for describing their use. The characteristics of some plants have not been fully studied to date. In particular, much research has been conducted in China, Egypt, India, Greece, and other countries [21]. Not all medicinal plants have the same effect on the human body. Medicinal plants have been used for thousands of years to flavor and conserve food, treat health disorders, and prevent diseases, including epidemics [22]. The knowledge of their healing properties has been transmitted over the centuries within and among human communities. These medicinal plants are considered a rich resource of ingredients that can be used in drug development and synthesis [23]. Some people are sensitive to medicinal plants, while others are allergic to them. This means that each person needs separate recommendations [20,24]. Despite the use of herbal medicines over many centuries, only a relatively small number of plant species have been studied as possible drugs [25,26]. The analysis of medicinal plants is considered important, as not all of them have a positive effect. Some negatively affect the body if consumed in large quantities [27]. Ethnobotanical and ethnopharmacological studies increase the possibility of identifying new molecules instead of random screening [28,29]. Plants are threatened in many parts of the world. According to IUCN [30] data, 17,000 species of medicinal plants are globally threatened due to, amongst other factors, the loss of habitat, overexploitation, invasive species, and pollution [30,31].

Medicinal plants are diverse, and they can have anti-diabetic properties. They also have distinctive effects on the human body [32]. Central Asia is rich in plants that are widely used by locals in the traditional way, especially in Uzbekistan and Kyrgyzstan, where the population has a lot of knowledge regarding their use [33]. This is possibly due to the difficulties that these populations face regarding their current health systems [34].

Much work has been conducted on the conservation and use of medicinal plants [35], as well as on their commercialization [29,36,37]. In recent years, much scientific work has also been undertaken on the connections between traditional and modern medicines [38,39].

Much research has been conducted in Uzbekistan on the study of species with medicinal and economic importance [40]. At the same time, the populations of some medicinal plants in the desert region were studied [41].

In Uzbekistan, the use of natural resources is considered one of the true economic sources for the Republic of Uzbekistan. The development of the national economy of the Republic depends on the degree of literacy and the rational use of natural resources [42]. Biological resources, including medicinal plants, are the most important component of the natural wealth of Uzbekistan. To determine the current state of medicinal plants in order to enhance their subsequent protection and sustainable use, it is necessary to obtain annual information on the state of their distribution, their population size, and the impact of negative factors on their populations [43].

**Figure 1:**
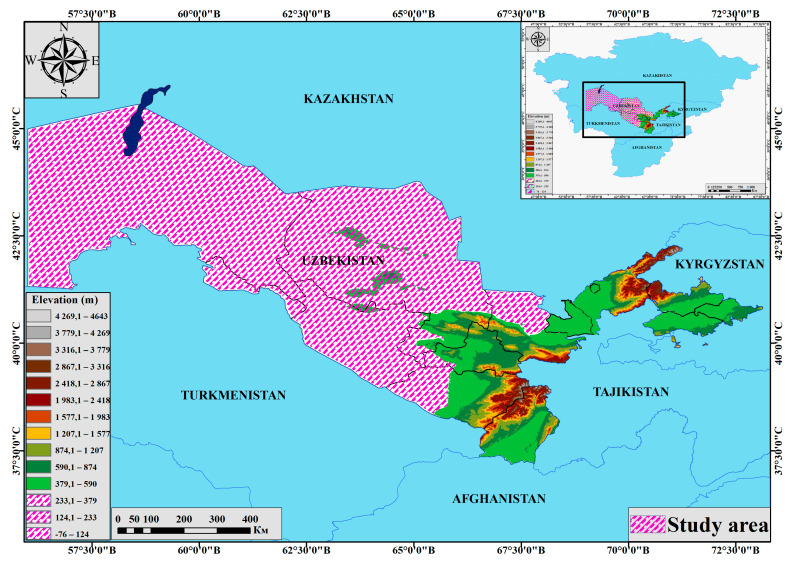
Map of the study area.

In this study, we analyzed and sorted the collection records resulting from our fieldwork conducted during the period from 2012 to 2022 (Figure 1) and checked them against published records in the literature to update the checklists of the main medicinal plants in the arid regions of Uzbekistan, to determine the species composition of medicinal plants distributed in the arid regions of Uzbekistan, and to characterize the traditional use of some plants in folk medicine.

## 2. Results

Ethnobotany can be defined as plant–human relations, which have existed throughout human history [44,45]. In the study, 529 medicinal species belonging to 70 families and 269 genera were identified in the study region (Table S1). The arid region of Uzbekistan is unique in having an annual precipitation of around 100–150 mm. In some years, this amount does not exceed 80 mm. The medicinal plants of the region are also not evenly distributed. While the distribution of some plants is very wide, some grow in very small areas. The presence of regular strong winds in desert conditions negatively affects the reproduction of medicinal plants, even in nurseries. Livestock grazing in these regions throughout the year also has a negative impact on plant populations. Through studies, the percentages of medicinal plant species belonging to different families were calculated. According to these studies, representatives of 70 families are distributed in the arid regions of Uzbekistan. Each family comprises between 0.18 and 13.79% of the total species in the area (Table 1).

**Figure 2:**
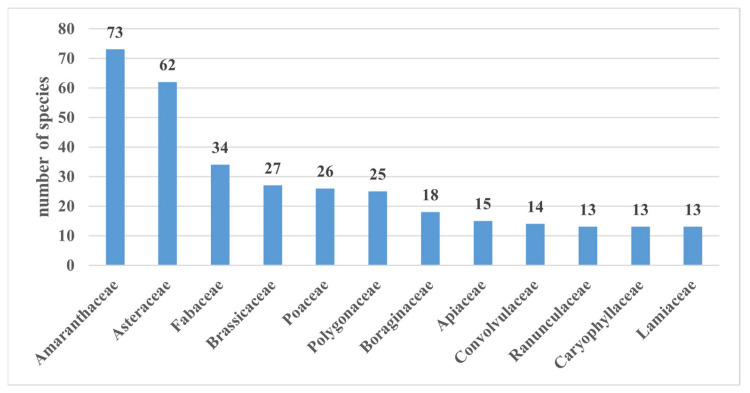
Most representative families.

During the studies, an analysis of 12 large families was carried out. The number of species in these families ranged from 13 to 73. The family Amaranthaceae comprised the largest number of species, with 73. This situation is typical for the arid regions of Central Asia since representatives of this family are well adapted to growing on salty and arid lands. The next most represented families were Asteraceae with 62 species; Fabaceae with 34 species; Brassicaceae with 27 species; Poaceae with 26 species; Polygonaceae with 25 species; Boraginaceae with 18 species; Apiaceae with 15 species; Convolvulaceae with 14 species; and Ranunculaceae, Caryophyllaceae, and Lamiaceae, each with 13 species. These families made up 62.94% of the total species (Figure 2).

The remaining families accounted for 37.06% of the total. The families Ixioliriaceae, Elaeagnaceae, Cucurbitaceae, Dryopteridaceae, Juncaginaceae, Utricaceae, Portulacaceae, Verbenaceae, Hippuridaceae, Mazaceae, Primulaceae, Araceae, Vitaceae, and Biebersteiniaceae were represented in the flora by only one species each.

**Figure 3:**
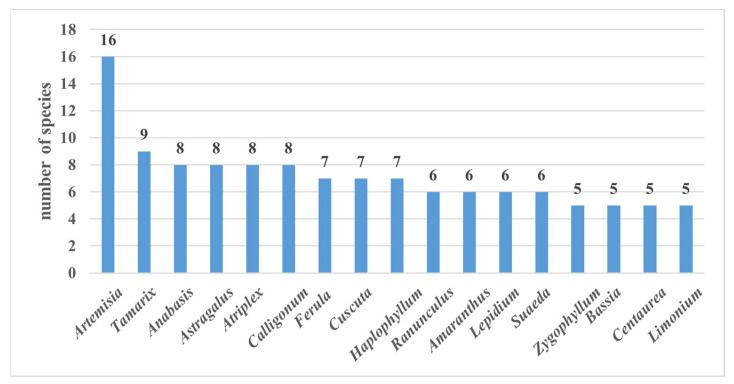
Most representative genera.

During the studies, an analysis of the medicinal plants by genus was carried out. The 17 most prevalent medicinal plant genera in the region were identified. The number of species in each genus ranged from five to sixteen, with the largest number of species determined for *Artemisia* (16). The next most represented genera were *Tamarix* with nine species; *Anabasis*, *Astragalus*, *Atriplex*, and *Calligonum*, each with eight species; *Ferula*, *Cuscuta*, and *Haplophyllum*, each with seven species; *Ranunculus*, *Amaranthus*, *Lepidium*, and *Suaeda*, each with six species; and *Zygophyllum*, *Bassia*, *Centaurea*, and *Limonium*, each with five species (Figure 3).

Representatives of *Artemisia* are widespread in the arid regions of Uzbekistan. This genus comprises 39 species distributed in the flora of Uzbekistan, and these species are the main essential oil-producing plants. At the same time, these species are the main feed for livestock. *Artemisia diffusa*, *A turanica*, and *A. sogdiana* form large areas in the arid regions of Uzbekistan.

Species of *Tamarix* are abundant in the wetlands of arid regions, as well as in areas where moisture is preserved. They are also adapted to the external environment and to growing on brackish land. Representatives of this genus are also considered frost-resistant. According to representative members of the local population, the species in this category contain a lot of honey juice.

Representatives of *Ferula* are also widely used in a traditional way in the countries of Central Asia. These plants are widely used, mainly to treat internal diseases. In the arid regions of Uzbekistan, seven medicinal species of this genus grow. In some years, they dominate the vegetation cover.

*Calligonum* species are also common in the desert and have many characteristics. In particular, they are widely used for combating desertification, as well as for medicinal treatments.

## 3. Discussion

The flora of Uzbekistan contains more than 4500 vascular plants belonging to 1002 genera in 171 families [46]. More than 4000 species of algae and more than 2000 species of fungi also occur in Uzbekistan. The flora is dominated by Asteraceae (600 species), Fabaceae (450 species), Poaceae (>250 species), Brassicaceae, Lamiaceae, Rosaceae, Boraginaceae, and Apiaceae.

During the study, plants common to the arid regions of Uzbekistan were studied. These species are widespread in most parts of the region and occupy large areas. In the region, *Peganum harmala* L., *Capparis spinosa* L., *Ferula foetida* (Bunge) Regel, *Glycyrrhiza glabra* L., *Alhagi pseudalhagi*, *Lagochilus inebrians* Bunge, *Xanthium strumarium* L., *Silybum marianum* (L.) Gaerth., *Onopordom acanthium* L., *Ziziphora tenuior* L., and *Cichorium intybus* L. species cover a large area and have been used regularly by the locals since ancient times.

**Figure 4:**
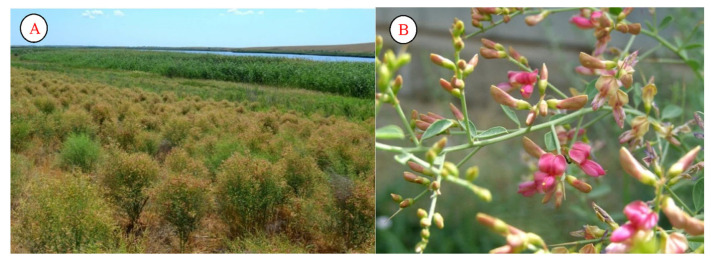
Population (**A**) and flower (**B**) of *Alhagi pseudalhagi* (lake Aydarkul).

During the research, semi-structured interviews were conducted with tabibs, elders, herders, and residents with experience in healing using medicinal plants. We spoke to them about the main medicinal plants distributed in the arid regions of Uzbekistan. *Alhagi pseudalhagi* is common in the region. A huge amount of honey can be obtained from the plant. This species blooms in Uzbekistan during May–September. For livestock, too, the fruit of the plant is a good nutrient. It is used in local medicine for the treatment of internal diseases, as well as in the preparation of good tinctures to protect against sunlight exposure (Figure 4).

**Figure 5:**
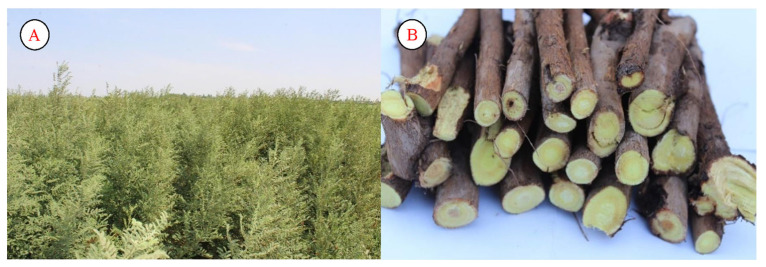
Population (**A**) and roots (**B**) of *Glycyrrhiza glabra* (Khorezm region).

*Glycyrrhiza glabra* is mainly distributed in the regions of Khorezm and Karakalpakstan, Uzbekistan. This species is resistant to saline soils and is mainly distributed close to water bodies, as well as in areas with high humidity. The species grows well mainly in areas where groundwater is nearby. Its underground part is used very widely by the local population. In modern medicine, glyciram, glycirama, lycviriton, and flacarbin are widely used to obtain the corresponding drugs. Local residents use the plant for urinary and cough disorders. Under Uzbek conditions, the plant blooms in May–June. In August–September, the fruits ripen completely (Figure 5).

**Figure 6:**
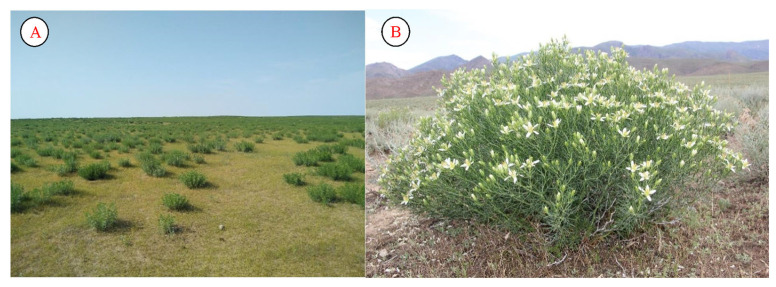
Population (**A**) and flower (**B**) of *Peganum harmala* (eastern Kyzylkum).

*Peganum harmala* is used as a healing plant to treat thousands of diseases. It is very common in arid regions of Uzbekistan. The plant is adapted to a variety of ecological environments. Even as a result of the degradation of deserts, the range of this species has expanded. This species is also found around swampy lands. It contains a large number of alkaloids. It is also widely used in the treatment of internal diseases. The stem has been widely used by locals for several centuries. It is widely used in all Central Asian countries. The seeds have characteristics that allow them to be used to treat pus, toothaches, sweating, and diseases of the gastrointestinal system, and to prevent the flu (Figure 6).

In Tajikistan (which borders Uzbekistan), smoke from burning the plant is used to treat paralytics. The leaves are used as a poultice to treat swelling [47]. In scientific medicine, the above-ground part of the plant is used. Its seeds contain 3.5–6.0% of the plant’s alkaloids; 60% of its garmalin; 30% of its garmin; small amounts of garmalol, peganin, and deoxyribose; and 60% of the plant’s peganin (vasicina) and vasicinones, which contain 1.5–3% of the alkaloids in the plant.

**Figure 7:**
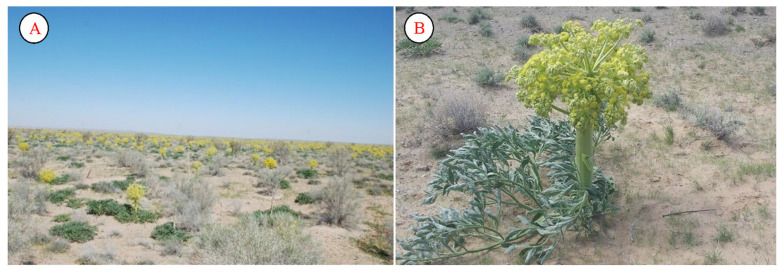
Population (**A**) and individual (**B**) of *Ferula foetida*.

In recent years, much work has also been carried out by scientists from the Institute of Botany of the Academy of Sciences of the Republic of Uzbekistan on the identification and use of reserve *Ferula foetida* (Figure 7). The 4–5-year-old vegetation of this species provides good aphid control. The plant is a monocarp and blooms only once in its life. It is widespread in mainly sandy soils throughout Uzbekistan. An herbal decoction made from this plant has analgesic and calming properties. The plant also contains essential oil. In addition, anti-parasitic drugs have been prepared from this plant for use in young children. In Uzbek and Tajik medicine, the young leaves of the plant are mixed with water to treat homeopathy, syphilis, and tumors; in India, the resin is extracted for use in scientific medicine and folk medicine; and in Japan, Paraguay, Portugal, Turkey, Italy, China, Spain, and France, the plant is used for the treatment of neurosis, bod, bronchial asthma, skin inflammation, hematomas, malignant tumors (tumors), pulmonary tuberculosis, and other diseases. In recent years, a lot of work has also been carried out on the genomic analysis of representatives of the genus, as well as on the justification of their medicinal properties [48].

*Capparis spinosa* is common in the rocky–gravelly soils of Uzbekistan. The species grows extensively, mainly on roadsides. It is highly resistant to external environmental influences and is not eaten by livestock. Its fruit is widely used, mainly in the preparation of salads.

**Figure 8:**
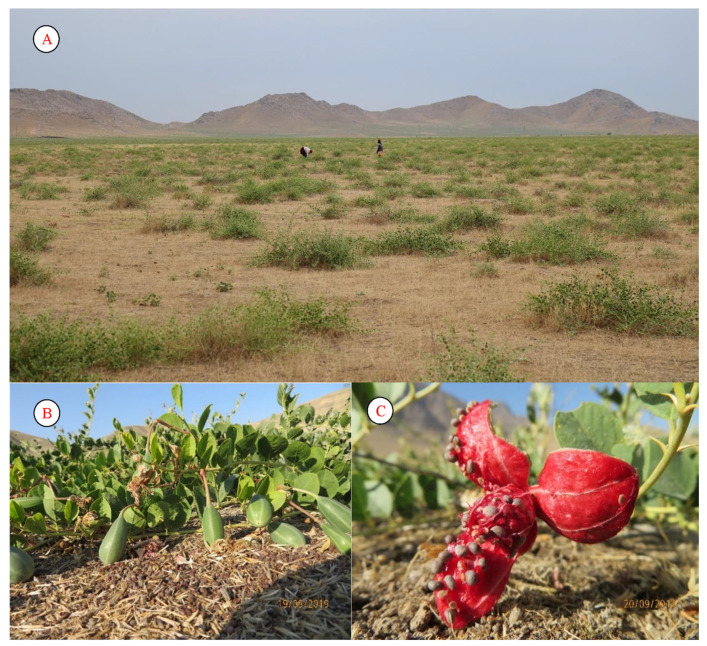
Population (**A**), fruits (**B**), and seeds (**C**) of *Capparis spinosa*.

An adult *C. spinosa* does not need surface moisture at all, does not suffer from drought, and can grow where other plants die. The species is distributed throughout the territory of Uzbekistan and Central Asia; it is found mainly in desert and semi-desert zones, foothills, and low mountains, sometimes reaching the middle belt of mountains. It can often be found in destroyed buildings and mosques. In some places, this species forms almost pure overgrowths over very large areas, especially in the arid (foothill) zone of the Republic of Uzbekistan (Figure 8).

Traditional medicine has used capers for a long time and very actively. All parts of this plant are used by healers to combat ailments such as hypertension, scabies, jaundice, neurosis, and brucellosis. Even the ancient Arabs cured all kinds of allergies and rheumatism with the help of the roots of capers. The unblown buds of capers are an excellent appetite stimulant (Figure 8).

**Figure 9:**
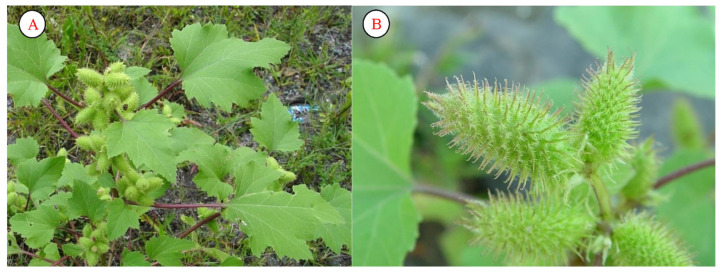
Individual (**A**) and fruits (**B**) of *Xanthium strumarium*.

*Xanthium strumarium*, as a weed, is widespread in the arid regions of Uzbekistan. Under Uzbek conditions, the plant blooms in July–September (Figure 9). Many scientists have conducted their own research on the importance and use of this species worldwide. These studies have achieved a huge number of scientific results [49,50,51,52].

**Figure 10:**
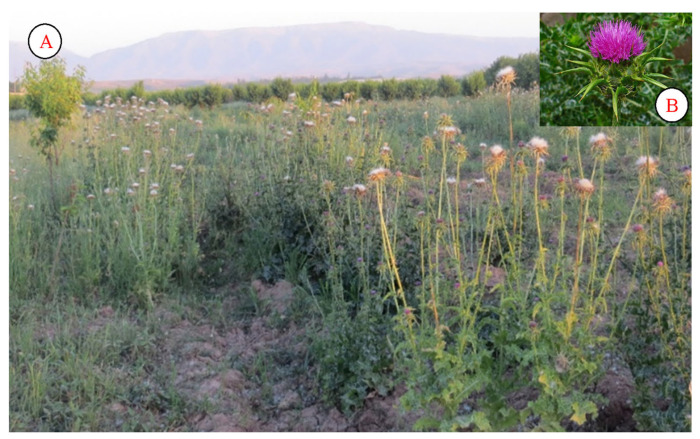
Population (**A**) and flower (**B**) of *Silibum marianum*.

*Silibum marianum* is mainly characterized as a ruderal plant in the flora of Uzbekistan. The plant is highly resistant to external environmental factors. It is also not eaten by livestock. This allows it to be more widely distributed in nurseries. It blooms in May in the arid regions of Uzbekistan. Local residents use herbal decoctions to treat liver cirrhosis and hepatitis. Much research has also been conducted on this plant by local scientists [47,53,54]. At the end of the last century, Gammerman et al. [55] and Sonnenbichler et al. [56] obtained many scientific results. Nowadays, this plant is widely grown for use in modern medicine (Figure 10).

**Figure 11:**
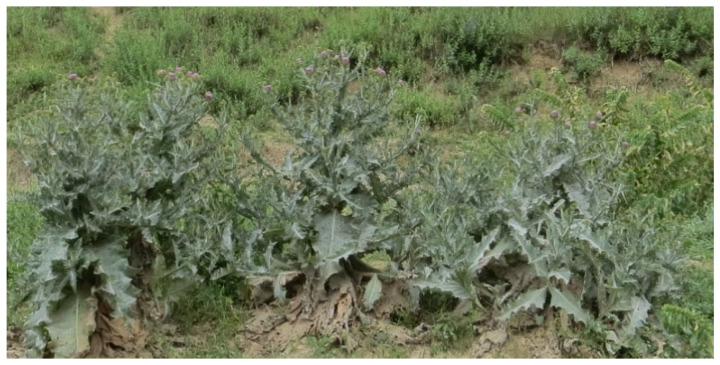
Population of *Onopordum acanthium*.

In Uzbekistan, the flower of *Onopordum acanthium* is used to accelerate wound healing (Figure 11). The plant’s distribution is similar to that of the *Silibum marianum* species, and it is highly tolerant to external environmental factors. It is mainly found on roadsides as well as in open areas. It is also abundant in some agricultural fields. It is used for the treatment of infectious diseases in the human body. Tinctures and decoctions made from the aboveground part are used to improve the activity of the central nervous system and increase strength [57]. In Kazakh folk medicine, it is used to treat bronchial asthma, tuberculosis, colds, skin diseases, headaches, infectious diseases, and shock [57].

Eight flavonoids were identified from *O. acanthium* by foreign scientists, in addition to two phenolic carbonic acids [58], two lignans [59], lactone, succinic acid, and the organic compound choline [58]. In Uzbekistan, N.T. Ulchenko and E.I. Gigienova studied the oils of *Onopordum acanthium*. The chemical composition of cotton thistle is poorly understood. Due to its rich chemical composition, this plant has many beneficial properties and is widely used in medicine, the perfume industry, and cooking [60].

**Figure 12:**
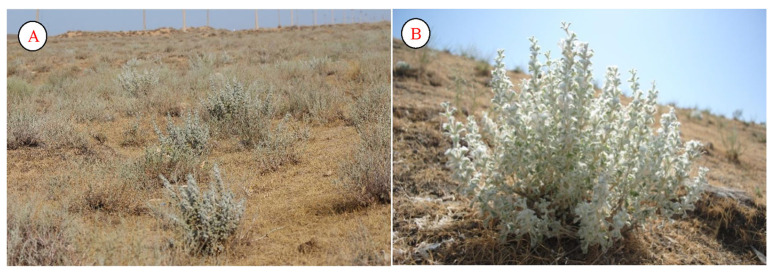
Population (**A**) and individual (**B**) of *Lagochilus inebrians* (Kyzykum desert).

The range of *Lagochilus inebrians* is not very large. Locals widely use this plant, mainly as tea. It grows as a community in some areas. The plant contains many substances necessary for the human body [61]. As the most common species of its genus, *L. inebrians* grows in the Samarkand, Bukhara, Kashkadarya, and Surkhandarya regions of the Republic of Uzbekistan (Figure 12).

Considering the medicinal properties of the species, in the years 1970–1980 and the vicinity of the city of Arys in the Chimkent region of Kazakhstan, large plantations of *L. inebrians* were organized on the Darmin farm. Currently, to preserve the natural population of the species and to obtain raw materials, it is cultivated in the Tashkent, Samarkand, and Navoi regions of the Republic of Uzbekistan. *L. inebrians* contains the diterpene alcohol lagochilin, mono- and diacetyl derivatives of lagochilin, vulgarol, essential oils, tannins, and resinous substances, carotene, vitamins K and C, organic acids, calcium, magnesium, and 20 different microelements [62]. As the authors point out, *L. inebrians* has a wide range of therapeutic effects. It is used in folk and traditional medicine as a hemostatic agent in the form of infusions and tinctures. However, the use of the infusions and tinctures of the intoxicating lagochilus plant is associated with several inconveniences: the instability of the infusion, inaccurate dosages, and a bitter taste. All these drugs are of low toxicity; however, the main disadvantage of all such drugs is that they can only be administered orally since intravenous administration is unacceptable.

**Figure 13:**
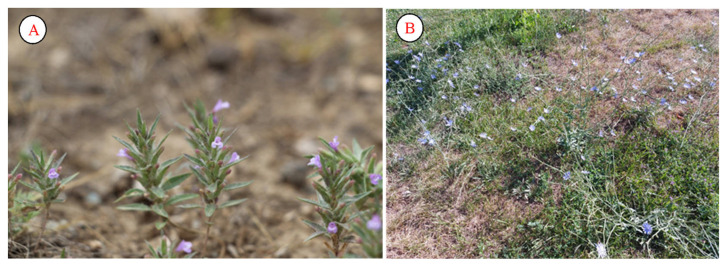
Ziziphora tenuior (**A**) and Cichorium intybus (**B**).

In addition, many plants are drunk in the state of tea to stabilize blood pressure. Examples include *Ziziphora tenuior* (Figure 13A) and *Cicorium intybus* (Figure 13B). Local people use *Ziziphora tenuior* very widely. In almost all families, the *Cicorium intybus* plant is used as a tea to lower blood pressure. *Cicorium intybus* is a valuable medicinal plant that has long been popular in folk medicine. The roots were used in ancient Rome to improve digestion; in Egypt, the plant was used to prepare an anti-venom serum for the venom of snakes and spiders. The famous Abu Ali ibn Sina used splashes to treat diseases of the gastrointestinal tract and eyes, inflammation of the eyes, and gout. In modern medicine, sachratqi has a wide variety of applications due to its beneficial medicinal properties (can be used to lower sugar levels, reduce fevers, and treat urinary disorders and vomiting; also possesses binding, anti-inflammatory, and soothing properties).

## 4. Materials and Methods

### 4.1. Study Area

Although the bulk of Uzbekistan consists of arid regions, mountain passes are also found in this region. Mountain forests make up 15–17% of the total area. The main part of these areas is widely used as pastures. The height of the territory above sea level is 50–600 m (Figure 1).

### 4.2. Plant Materials

The study was carried out during the period from 2012 to 2022. In this study, samples collected in the field, as well as samples held at the Herbarium (TASH) of the Institute of the Botany Academy of Sciences, Uzbekistan, were analyzed. The plant species indicated as useful by respondents were collected, pressed, dried, mounted, and preserved based on the standard methods given in [63]. Species identifications as well as an analysis of their occurrences were made using the classical literature of V.L. Komarov [64], A.N. Sennikov [65], N.V.Pavlov [66], P.N.Ovchinnikov [67], B.A. Fedtscihenko et al. [68], and Shishkin and Vvedensky [69]. Before preservation, all the collected vouchers were examined and identified with the help of the literature [46]. Furthermore, the species were confirmed by comparing the samples with herbarium specimens deposited at the TASH (National Herbarium Institute of Botany Academy Sciences of the Republic of Uzbekistan); the Laboratory of Geobotany and Plant Ecology Institute of Botany Academy Sciences, Republic of Uzbekistan; and the CAS Key Laboratory of Biogeography and Bioresources in Arid Land, Xinjiang Institute of Ecology and Geography, China. The scientific name of plants and their families were verified by referring to the POWO [70].

### 4.3. The Traditional Use Surveys

The surveys on the traditional use of medicinal plants were conducted with residents of different ages. In the interviews, information was mainly received on the interviewee’s age, gender, diseases, and the plants that were used to fight their diseases. At the same time, information on plants previously used for traditional healing was received from the older population.

## 5. Conclusions

Medicinal plants and their uses are considered very important to human life. In recent years, many scientific studies on medicinal plants have been carried out. The results of this study represent the conclusions of long-term scientific work. In this study, the checklists of the main medicinal plants in the arid regions of Uzbekistan were updated; it was found that the region contains 529 species belonging to 70 families and 269 genera. Amaranthaceae (73) and *Artemisia* (16) are the largest family and genus, respectively. Several species, including *Peganum harmala* L., *Capparis spinosa* L., *Ferula foetida* (Bunge) Regel, *Glycyrrhiza glabra* L., *Alhagi pseudalhagi* (M.Bieb.) Desv. ex Wangerin, *Lagochilus inebrians* Bunge, *Xanthium strumarium* L., *Silybum marianum* (L.) Gaerth., and *Onopordom acanthium* L., cover a large area and have been widely used by representative members of the local population in folk medicine for several years. The obtained results serve as key data in the field of pharmaceutics and provide an opportunity to monitor species distributions and study the population dynamics of medicinal plants.

## Figures and Tables

**Table 1:** Medicinal plants distributed in the arid regions of Uzbekistan.

№	Family	Genus	Species	Percentage of Species, %
1	Equisetaceae	1	2	0.37
2	Ephedraceae	1	4	0.75
3	Colchicaceae	1	2	0.37
4	Ixioliriaceae	1	1	0.18
5	Asparagaceae	1	4	0.75
6	Juncaceae	1	5	0.94
7	Cyperaceae	4	10	1.89
8	Poaceae	22	26	4.91
9	Asphodelaceae	1	2	0.37
10	Amaryllidaceae	1	2	0.37
11	Typhaceae	1	3	0.56
12	Papaveraceae	1	3	0.56
13	Fumariaceae	3	3	0.56
14	Berberidaceae	1	2	0.37
15	Ranunculaceae	5	13	2.45
16	Zygophyllaceae	2	7	1.32
17	Fabaceae	17	34	6.43
18	Rosaceae	4	9	1.70
19	Elaeagnaceae	1	1	0.18
20	Cucurbitaceae	1	1	0.18
21	Euphorbiaceae	3	9	1.70
22	Lythraceae	2	3	0.56
23	Onagraceae	2	4	0.75
24	Nitrariaceae	3	4	0.75
25	Rutaceae	1	7	1.32
26	Thymelaeaceae	2	2	0.37
27	Malvaceae	4	7	1.32
28	Capparaceae	1	2	0.37
29	Frankeniaceae	1	2	0.37
30	Dryopteridaceae	1	1	0.18
31	Juncaginaceae	1	1	0.18
32	Moraceae	1	2	0.37
33	Salicaceae	2	4	0.75
34	Urticaceae	1	1	0.18
35	Caprifoliaceae	3	3	0.56
36	Tamaricaceae	1	9	1.70
37	Plumbaginaceae	1	5	0.94
38	Brassicaceae	19	27	5.10
39	Polygonaceae	7	25	4.72
40	Caryophyllaceae	6	13	2.45
41	Amaranthaceae	32	73	13.79
42	Portulacaceae	1	1	0.18
43	Rubiaceae	2	6	1.13
44	Gentianaceae	3	4	0.75
45	Apocynaceae	3	3	0.56
46	Boraginaceae	12	18	3.40
47	Convolvulaceae	4	14	2.64
48	Solanaceae	5	9	1.70
49	Plantaginaceae	2	5	0.94
50	Scrophulariaceae	3	6	1.13
51	Verbenaceae	1	1	0.18
52	Lamiaceae	10	13	2.45
53	Hippuridaceae	1	1	0.18
54	Mazaceae	1	1	0.18
55	Orobanchaceae	2	6	1.13
56	Asteraceae	29	61	11.53
57	Apiaceae	9	15	2.83
58	Primulaceae	1	1	0.18
59	Geraniaceae	2	3	0.56
60	Butomaceae	1	1	0.18
61	Alismataceae	2	4	0.75
62	Potamogetonaceae	2	4	0.75
62	Potamogetonaceae	2	4	0.75
63	Lemnaceae	1	2	0.37
64	Araceae	1	1	0.18
65	Iridaceae	1	2	0.37
66	Liliaceae	1	2	0.37
67	Rhamnaceae	2	2	0.37
68	Ulmaceae	1	2	0.37
69	Vitaceae	1	1	0.18
70	Biebersteiniaceae	1	1	0.18
Total	70	269	529	100%

## Data Availability

The data presented in this study are available.

## Supplementary Materials

The following supporting information can be downloaded at: https://www.mdpi.com/article/10.3390/plants12162950/s1. Table S1: checklist of medicinal plants distributed in the arid regions of Uzbekistan.
