# Supplementary material for: The Main Medicinal Plants in Arid Regions of Uzbekistan and Their Traditional Use in Folk Medicine
Source: Plants (Basel). 2023 Aug 15;12(16):2950. doi: 10.3390/plants12162950 (PMC10458710; doi:10.3390/plants12162950)
Supplement: Supplementary file 1 [file plants-12-02950-s001.zip › plants-2322156-supplementary.pdf]

Table S1. Chenklist of medicinal plants distributed in the arid regions of Uzbekistan.

№	Plant Name
1.	**ALISMATACEAE VENT.**<br>Alisma lanceolatum With.
2.	Alisma plantago-aquatica L.
3.	Sagittaria sagittifolia L.
4.	Sagittaria trifolia L.
5.	**APOCYNACEAE JUSS.**<br>Trachomitum scabrum (Russanov) Pobed.
6.	*Cynanchum acutum* subsp. *sibiricum* (Willd.) Rech. f.
7.	Vincetoxicum sibiricum (L.) Decne.
8.	**ARACEAE JUSS.**<br>Eminium lehmannii (Bunge) O.Kuntze
9.	**AMARANTHACEAE JUSS.**<br>Agriophyllum lateriflorum (Lam.) Moq.
10.	Agriophyllum latifolium Fisch. & C.A.Mey.
11.	Agriophyllum minus Fisch. & C.A.Mey.ex Fenzi
12.	Amaranthus albus L.
13.	Amaranthus blitoides S.Watson
14.	Amaranthus blitum L.
15.	Amaranthus graecizans L.
16.	Amaranthus hybridus L.
17.	Amaranthus retroflexus L.
18.	Anabasis annua Bunge
19.	Anabasis aphylla L.
20.	Anabasis brachiata Fisch. & C.A. Mey. ex Kar. & Kir.
21.	Anabasis cretacea Pall.
22.	Anabasis eriopoda (Schrenk) Paulsen
23.	Anabasis salsa (Ledeb.) Benth. ex Volkens
24.	*Anabasis truncata* (Schrenk) Bunge
25.	Anabasis turkestanica Korovin ex Iljin
26.	Atriplex prostrata subsp.calotheca (Rafn) M.A.Gust.
27.	Atriplex micrantha Ledeb.
28.	Atriplex moneta Bunge ex Boiss.
29.	Atriplex patula L.
30.	Atriplex prostrata Boucher ex DC.
31.	Atriplex sagittata Borkh.
32.	Atriplex sibirica L.
33.	Atriplex tatarica L.
34.	Bassia hyssopifolia (Pall.) Kuntze
35.	Bassia odontopera (Schrenk) Freitag& G.Kadereit
36.	Bassia prostrata (L.) Beck
37.	Bassia Pilosa (Fisch.& C.A.Mey) Freitag&G.Kaderit
38.	Bassia scoparia (L.) F.J.Scott
39.	Blitum virgatum L.
40.	Camphorosma lessingii Litv.
41.	Camphorosma monspeliaca L.
42.	Caroxylon dendrides (Pall.) Tzvelev
43.	Caroxylon gemmascens (Pall.) Tzvelev

44.	Ceratocarpus arenarius L.
45.	Chenopodium album L.
46.	Chenopodiastrum murale (L.) S.Fuentes, Uotila&Borsch
47.	Chenopodium vulvaria L.
48.	Climacoptera transoxana<br>(Iljin) Botsch.
49.	Corispermum lehmannianum Bunge
50.	Corispermum squarrosum L.
51.	*Dysphania botrys* (L.) Mosyakin et Clemants
52.	Gamanthus gamocarpus (Moq.) Bunge
53.	Girgensohnia oppositiflora (Pall.) Fenzl
54.	Halocharis hispida (Schrenk) Bunge
55.	Halocnemum strobilaceum (Pall.) M.Bieb.
56.	Halostachys casica (M.Bieb) C.A.Mey.
57.	Halothamnus subaphyllus (C.A. Mey.) Botsch
58.	Halothamnus glaucus (Bieb.) Botsch.
59.	Halothamnus iliensis (Lipsky) Botsch.
60.	Haloxylon ammodendron<br>(C.A.Mey.) Bunge ex Fenzl
61.	Haloxylon persicum Bunge
62.	Kalidium caspicum (L.) Ung.-Sternb.
63.	Krascheninnikovia ceratoides (L.) Gueldenst.
64.	Nanophyton erinaceum (Pall.) Bunge
65.	Oxybasis rubra (L.) S.Fuentes, Uotila&Borsch
66.	Oxybasis urbica (L.) S.Fuentes, Uotila&Borsch
67.	Salicornia europaea L.
68.	Salsola australis R. Br.
69.	Salsola collina Pall.
70.	Soda inermis Fourr.
71.	*Spinacia turkestanica* Iljin
72.	Suaeda altissima (L.) Pall.
73.	Suaeda arcuata Bunge
74.	Suaeda lehmannii (Bunge) Karpalov, Akhani &Roalson
75.	Suaeda microphylla Pall.
76.	Suaeda physophora Pall.
77.	Suaeda prostrate Pall.
78.	Turania sogdiana (Bunge) Akhani
79.	Xylosalsola arbuscular (Pall.) Tzvelev
80.	Xylosalsola paletzkiana (Litv.) Akhani &Roalson
81.	Xylosalsola richteri (Moq.) Akhani &Roalson
82.	**AMARYLLIDACEAE.**<br>Allium caeruleum Pall
83.	Allium sabulosum Steven ex Bunge
84.	**APIACEAE LINDL.**<br>Chaerophyllum prescottii DC.
85.	Cuminum setifolium (Boiss.) Koso-Pol.
86.	*Daucus carota* L.
87.	Dorema sabulosum Litv.
88.	*Eryngium octophyllum* Korovin
89.	Ferula caspica M. Bieb.

90.	*Ferula diversivittata* Regel & Schmalh.
91.	Ferula dubjanskyi Korovin
92.	Ferula foetida (Bunge) Regel
93.	Ferula karelinii Bunge
94.	*Ferula litwinowiana* Koso-Pol.
95.	Ferula varia (Schrenk) Trautv.
96.	Scandix pecten-veneris L.
97.	Seseli jomuticum Schischk.
98.	Zosima absinthiifolia (Vent.) Link
99.	**ASPARAGACEAE JUSS.**<br>Asparagus brachyphyllus Turcz.
100.	Asparagus officinalis L.
101.	Asparagus persicus Baker
102.	Asparagus turkestanicus Popov
103.	**ASPHODELACEAE JUSS.**<br>Eremurus anisopterus (Kar. & Kir.) Regel
104.	Eremurus inderiensis (M.Bieb.) Regel
105.	**ASTERACEAE BERCHT. & J.PRESL**<br>Achillea nobilis L.
106.	*Achillea santolinoides* subsp. *wilhelmsii* (K. Koch) Greuter
107.	*Achillea arabica* Kotschy
108.	Acroptilon repens (L.) DC.
109.	Amberboa nana (Boiss.) Ilgin
110.	Amberboa turanica Ilgin
111.	Artemisia annua L.
112.	Artemisia arenaria DC.
113.	Artemisia austriaca Jacq.
114.	Artemisia biennis Wild.
115.	Artemisia diffusa Krasch.ex Poljakov
116.	*Artemisia ferganensis* Krasch. ex Poljakov
117.	Artemisia juncea Kar. & Kir.
118.	Artemisia leucodes Schrenk
119.	Artemisia oliveriana J.Gay ex Besser
120.	Artemisia pauciflora Weber ex Stechm
121.	Artemisia santolina Schrenk
122.	Artemisia scoparia Waldst. & Kit.
123.	Artemisia songarica Schrenk ex Fisch.&C.A.Mey
124.	Artemisia terrae-albae Krasch.
125.	Artemisia turanica Krasch.
126.	Artemisia vulgaris L.
127.	Bidens tripartita L.
128.	Centaurea benedcta (L.)L.
129.	Centaurea depressa M.Bieb.
130.	*Centaurea iberica* Trevir. ex Spreng
131.	*Centaurea pulchella* Ledeb.
132.	*Centaurea virgata* subsp. *squarrosa* (Boiss.) Gugler
133.	Cichorium intybus L.
134.	*Cirsium arvense* (L.) Scop.
135.	*Cirsium vulgare* (Savi) Ten.
136.	*Erigeron canadensis* L.

137.	Filago arvensis L.
138.	*Filago pyramidata* L.
139.	*Garhadiolus papposus* Boiss. & Buhse
140.	*Handelia trichophylla* Heimerl
141.	Helichrysum arenarium (L.) Moench.
142.	Jurinea cyanoides (L.) Reichenb.
143.	Koelpinia linearis Pall.
144.	*Lachnophyllum gossypinum* Bunge
145.	Lactuca serriola L.
146.	Lactuca tatarica (L.) C.A.Mey.
147.	Lactuca undulata Ledeb.
148.	Microcephala lamellata (Bunge) Pobed.
149.	Onopordum acanthium L.
150.	*Pentanema britannicum* (L.) D.Gut.Larr., Santos-Vicente, Anderb., E.Rico & M.M.Mart.Ort.
151.	*Pentanema caspicum* (F.K.Blum ex Ledeb.) G.V.Boiko, Korniy. & Mosyakin
152.	*Pseudohandelia umbellifera* (Boiss.) Tzvelev
153.	Pulicaria gnaphalodes (Vent.) Boiss.
154.	Pulicaria vulgaris Gaertn.
155.	Saussurea salsa (Pall. ex Pall.) Spreng.
156.	Scorzonera gageoides Boiss.
157.	Senecio subdentatus Ledeb.
158.	Sonchus asper (L.) Hill
159.	*Sonchus oleraceus* L.
160.	Tanacetum santolina C.Winkl.
161.	Taraxacum bicorne Dahlst.
162.	Taraxacum comitans Kovalevsk
163.	*Taraxacum officinale* F.H. Wigg.
164.	*Tripolium pannonicum* (Jacq.) Dobrocz.
165.	*Xanthium spinosum* L.
166.	Xanthium strumarium L.
167.	**BERBERIDACEAE JUSS.**<br>Leontice ewersmannii Bunge
168.	Leontice incerta Pall.
169.	**BIEBERSTEINIACEAE SCHNIZL.**<br>Biebersteinia multifida DC.
170.	**BORAGINACEAE JUSS.**<br>*Anchusa azurea* Mill.
171.	Arnebia decumbens (Vent.) Coss. & Kralik
172.	Asperugo procumbens L.
173.	Cynoglossum viridiflorum Pall. ex Lehm.
174.	*Echium biebersteinii* Lacaita
175.	Heliotropium arguzioides Kar. & Kir.
176.	Heliotropium dasycarpum Ledeb.
177.	Heliotropium ellipticum Ledeb.
178.	*Heliotropium micranthos* (Pall.) Bunge
179.	*Heliotropium sogdianum* Bunge
180.	*Heliotropium dasycarpum* subsp. *transoxanum* (Bunge) Akhani & Förther
181.	*Lappula squarrosa* (Retz.) Dumort.
182.	Lappula microcarpa (Ledeb.) Gürke

183.	*Onosma dichroantha* Boiss.
184.	Rindera tetraspis Pall.
185.	*Tournefortia sibirica* L.
186.	*Trigonotis peduncularis* (Trevir.) Benth. ex Hemsl.
187.	*Trichodesma incanum* (Bunge) A. DC.
188.	**BRASSICACEAE BURNETT**<br>Alyssum dasycarpum Stephan ex Willd.
189.	Alyssum turkestanicum Regel & Schmalh.
190.	*Barbarea vulgaris* R. Br.
191.	Brassica juncea (L.) Czern.
192.	Capsella bursa-pastoris (L.) Medik.
193.	Chorispora tenella (Pall.) DC.
194.	Conringia orientalis (L.) C.Presl
195.	Crambe edentula Fisch & C.A. Mey. ex Korsh.
196.	Descurainia sophia (L.) Webb ex Prantl
197.	*Eruca vesicaria* (L.) Cav.
198.	Erucastrum armoracioides (Czern. ex Turez.) Cruchet
199.	*Erysimum diffusum* Ehrh.
200.	*Erysimum quadrangulum* Desf.
201.	Goldbachia laevigata DC.
202.	Isatis tinctoria L.
203.	Lachnoloma lehmannii Bunge
204.	*Lepidium appelianum* Al-Shehbaz
205.	*Lepidium chalepense* L.
206.	*Lepidium draba* L.
207.	Lepidium latifolium L.
208.	Lepidium perfoliatum L.
209.	Lepidium ruderale L.
210.	Rorippa palustris (L.) Besser
211.	Sinapis arvensis L.
212.	Sisymbrium irio L.
213.	Sisymbrium loeselii L.
214.	*Thlaspi arvense* L.
215.	**BUTOMACEAE**<br>*Butomus umbellatus* L.
216.	**CAPPARACEAE JUSS.**<br>*Capparis spinosa* var. *herbacea* (Willd.) Fici
217.	*Capparis spinosa* L.
218.	**CAPRIFOLIACEAE JUSS.**<br>Cephalaria syriaca (L.) Schrad.
219.	Lonicera tatarica L.
220.	*Valeriana triplaris* (Boiss. & Buhse) Christenh. & Byng
221.	**CARYOPHYLLACEAE JUSS.**<br>Acanthophyllum elatius Bunge
222.	Acanthophyllum korolkowii<br>Regel & Schmalh.
223.	Acanthophyllum pungens<br>(Bunge) Boiss.
224.	Acanthophyllum stenostegium<br>Freyn

225.	Gypsophila paniculata L.
226.	Gypsophila perfoliata L.
227.	*Gypsophila vaccaria* (L.) Sm.
228.	Holosteum umbellatum L.
229.	Lepyrodiclis holosteoides (C.A. Mey.) Fenzl ex Fisch. & C.A. Mey.
230.	*Silene nana* Kar. & Kir.
231.	Silene viscosa (L.) Pers.
232.	Stellaria graminea L.
233.	Stellaria media (L.) C.Presl
234.	**COLCHICACEAE DC.**<br>Colchicum kesselringii Regel
235.	*Colchicum robustum* (Bunge) Stef.
236.	**CONVOLVULACEAE JUSS.**<br>Calystegia sepium (L.) R. Br.
237.	Convolvulus arvensis L.
238.	Convolvulus divaricatus Regel & Scmalh.
239.	Convolvulus fruticosus Pall.
240.	Convolvulus hamadae (Vved.) V.Petrov
241.	*Convolvulus dorycnium* subsp. *subhirsutus* (Regel & Schmalh.) Sa'ad
242.	Cressa cretica L.
243.	Cuscuta approximate Bab.
244.	Cuscuta campestris Yunck.
245.	Cuscuta chinensis Lam.
246.	*Cuscuta europaea* L.
247.	Cuscuta lehmanniana Bunge
248.	Cuscuta monogyna Vahl
249.	*Cuscuta pedicellata* Ledeb.
250.	**CUCURBITACEAE**<br>*Bryonia melanocarpa* Nabiev
251.	**CYPERACEAE JUSS.**<br>*Bolboschoenus maritimus* (L.) Palla
252.	*Bolboschoenus maritimus* subsp. *affinis* (Roth) T.Koyama
253.	Carex melanostachya M.Bieb. ex Willd.
254.	Carex riparia Curtis
255.	Cyperus fuscus L.
256.	Cyperus glomeratus L.
257.	Cyperus longus L.
258.	Cyperus rotundus L.
259.	*Schoenoplectus lacustris* (L.) Palla
260.	*Schoenoplectiella mucronata* (L.) J.Jung & H.K.Choi
261.	**DRYOPTERIDACEAE CHING**<br>Dryopteris filix-mas (L.) Schott
262.	**ELAEAGNACEAE JUSS.**<br>Elaeagnus angustifolia L.
263.	**EPHEDRACEAE DUMORT.**<br>Ephedra distachya L
264.	Ephedra intermedia Schrenk & C.A.Mey.
265.	Ephedra lomatolepis Schrenk
266.	Ephedra strobilacea Bunge
267.	**EQUISETACEAE RICH. EX DC.**

	Equisetum arvense L.
268.	Equisetum ramosissimum Desf.
269.	**EUPHORBIACEAE JUSS.**<br>*Andrachne telephioides* L.
270.	*Chrozophora tinctoria* (L.) A.Juss.
271.	Chrozophora sabulosa Kar. & Kir.
272.	*Euphorbia anisopetala* (Prokh.) Prokh.
273.	Euphorbia chamaesyce L.
274.	Euphorbia densa Schrenk
275.	Euphorbia falcate L.
276.	Euphorbia seguieriana Neck.
277.	Euphorbia turczaninowii Kar. & Kir.
278.	**FABACEAE LINDL.**<br>*Alhagi maurorum* subsp. *canescens* (Regel) Yakovlev
279.	*Alhagi maurorum* subsp. *kirghisorum* (Schrenk) Yakovlev
280.	*Alhagi maurorum* Medik.
281.	Ammodendron conollyi Bunge ex Boiss.
282.	Ammodendron eichwaldii Ledeb.&C.A.Mey
283.	Astragalus ammophilus Kar. & Kir.
284.	Astragalus chiwensis Bunge
285.	*Astragalus crenatus* Schult.
286.	*Astragalus erioceras* Fisch. & C.A.Mey. ex Ledeb.
287.	*Astragalus filicaulis* Fisch. & C.A.Mey. ex Ledeb.
288.	Astragalus flexus Fisch.
289.	Astragalus tribuloides Delile
290.	Astragalus villosissimus Bunge
291.	*Cullen drupaceum* (Bunge) Stirthon
292.	Caragana grandiflora DC.
293.	*Caragana halodendron* (Pall.) Dum.Cours.
294.	Eremosparton aphyllum (Pall.) Fisch. & C.A.Mey.
295.	Glycyrrhiza aspera Pall.
296.	Glycyrrhiza glabra L.
297.	Lotus corniculatus L.
298.	Medicago lupulina L.
299.	*Medicago orthoceras* (Kar. & Kir.) Trautv.
300.	Medicago sativa L.
301.	Melilotus albus Medik.
302.	Melilotus officinalis (L.) Lam.
303.	Meristotropis triphylla Fisch. & C.A. Mey.
304.	*Prosopis farcta* (Banks & Sol.) J.F.Macbr.
305.	Smirnowia turkestana Bunge
306.	*Sophora alopecuroides* L.
307.	*Sophora lehmannii* (Bunge) Yakovlev
308.	Sphaerophysa salsula (Pall.) DC.
309.	*Trifolium pratense* L.
310.	*Trifolium repens* L.
311.	*Vicia lathyroides* L.
312.	**FRANKENIACEAE DESV.**<br>Frankenia hirsuta L.
313.	Frankenia pulverulenta L.

314.	**FUMARIACEAE**<br>Corydalis schanginii (Pall.) B.Fedtsch.
315.	*Fumaria vaillantii* Loisel.
316.	*Hypecoum pendulum* L.
317.	**GENTIANACEAE JUSS.**<br>Centaurium meyeri (Bunge) Druce
318.	*Centaurium pulchellum* (Sw.) Hayek ex Hand.-Mazz., Stadlm., Janch. & Faltis
319.	*Gentiana olivieri* Griseb.
320.	*Schenkia spicata* (L.) G.Mans.
321.	**GERANIACEAE JUSS.**<br>Erodium cicutarium (L.) L' Her.
322.	Erodium oxyrhinchum M.Bieb.
323.	*Geranium linearilobum* DC.
324.	**HIPPURIDACEAE**<br>*Hippuris vulgaris* L.
325.	**IRIDACEAE JUSS.**<br>Iris songarica Schrenk
326.	Iris tenuifolia Pall.
327.	**IXIOLIRIACEAE NAKAI**<br>Ixiolirion tataricum (Pall.) Schult. & Schult.fil.
328.	**JUNCACEAE JUSS.**<br>Juncus articulatus L.
329.	*Juncus bufonius* L.
330.	*Juncus gerardii* Loisel.
331.	Juncus compressus Jacq.
332.	*Juncus ranarius* Songeon & E.P.Perrier
333.	**JUNCAGINACEAE RICH**.<br>*Triglochin palustris* L.
334.	**LAMIACEAE LINDL.**<br>*Lagochilus acutilobus* (Ledeb.) Fisch. & C.A.Mey.
335.	*Lagochilus gypsaceus* Vved.
336.	*Lagochilus inebrians* Bunge
337.	Lallemantia royleana (Benth.) Benth.
338.	*Lamium amplexicaule* L.
339.	Lycopus europaeus L.
340.	*Marrubium anisodon* K. Koch
341.	*Mentha longifolia* var. *asiatica* (Boriss.) Rech.f.
342.	Nepeta cataria L.
343.	*Nepeta olgae* Regel
344.	*Salvia spinosa* L.
345.	Scutellaria galericulata L.
346.	Ziziphora tenuior L.
347.	**LEMNACEAE**<br>*Lemna gibba* L.
348.	*Lemna minor* L.
349.	**LILIACEAE JUSS.**<br>Gagea afghanica A.Terracc.
350.	Gagea reticulate (Pall.) Schult. & Schult.f.
351.	**LYTHRACEAE J. ST.- HIL.**<br>Ammannia baccifera L.

352.	*Lythrum linifolium* Kar. & Kir.
353.	Lythrum salicaria L.
354.	**MALVACEAE JUSS.**<br>Abutilon theophrasti Medik.
355.	*Alcea nudiflora* (Lindl.) Boiss.
356.	*Alcea rhyticarpa* (Trautv.) Iljin
357.	Althaea armeniaca Ten.
358.	Malva neglecta Wallr.
359.	Malva pusilla Smith
360.	Malva sylvestris L.
361.	**MAZACEAE**<br>*Dodartia orientalis* L.
362.	**MORACEAE GAUDICH.**<br>Morus alba L.
363.	Morus nigra L.
364.	**NITRARIACEAE LINDL.**<br>Malacocarpus crithmifolius<br>**(**Retz.) C.A. Mey.
365.	Nitraria schoberi L.
366.	Nitraria sibirica Pall.
367.	Peganum harmala L.
368.	**ONAGRACEAE JUSS.**<br>Epilobium hirsutum L.
369.	Epilobium palustre L.
370.	Epilobium tetragonum L.
371.	Oenothera biennis L.
372.	**OROBANCHACEAE VENT.**<br>Cistanche salsa (C.A. Mey.) G.Beck
373.	*Cistanche flava* (C.A. Mey.) Korsh.
374.	Orobanche cernua Loefl.
375.	Orobanche coerulescens Stephan ex Willd.
376.	Orobanche cumana Wallr
377.	Orobanche aegyptiaca Pers.
378.	**PAPAVERACEAE JUSS.**<br>Papaver pavoninum Schrenk
379.	*Papaver dodecandrum* (Forssk.) Medik.
380.	*Papaver refractum* (DC.) K.-F.Günther
381.	**PLANTAGINACEAE JUSS.**<br>*Holzneria spicata* (Korovin) Speta
382.	Plantago lachnantha Bunge
383.	Plantago lanceolata L.
384.	Plantago major L.
385.	Plantago maritime L.
386.	**PLUMBAGINACEAE JUSS.**<br>Limonium gmelinii (Willd.) O. Kuntze
387.	*Limonium meyeri* (Boiss.) Kuntze
388.	Limonium myrianthum (Schren) Kuntze
389.	Limonium otolepis (Schrenk) Kuntze
390.	Limonium suffruticosum (L.) Kuntze
391.	**POACEAE BARNHART**

	Aeluropus lagopoides (L.) Thwaites
392.	Agropyron desertorum (Fisch. ex Link.) Schult.
393.	Avena fatua L.
394.	Bothriochloa ischaemum (L.) Keng
395.	Calamagrostis epigejos (L.) Roth
396.	Chloris virgata Sw.
397.	Cynodon dactylon (L.)Pers.
398.	Dactylis glomerata L.
399.	Digitaria ischaemum (Schreb.) Mueh
400.	Digitaria sanguinalis (L.)Scop.
401.	Echinochloa crus-galli (L.) Beauv.
402.	*Elymus repens* (L.) Gould
403.	Imperata cylindrica (L.) Beauv.
404.	Lolium multiflorum Lam.
405.	*Lolium perenne* L.
406.	*Lolium temulentum* L.
407.	Phalaris arundinacea (L.)
408.	Phragmites australis (Cav.) Trin. ex Steud.
409.	Poa annua L.
410.	Poa bulbosa L.
411.	Saccharum spontaneum L.
412.	Setaria pumila (Poir.) Roem. & Schult.
413.	*Sorghum halepense* (L.) Pers.
414.	Stipa capillata L.
415.	Stipagrostis pennata (Trin.) De Winter
416.	*Thinopyrum intermedium* (Host) Barkworth & D.R.Dewey
417.	**POLYGONACEAE JUSS.**<br>Atraphaxis frutescens (L.) K. Koch.
418.	Atraphaxis spinosa L.
419.	Calligonum aphyllum (Pall.) Guerke
420.	Calligonum arborescens Litv.
421.	Calligonum caput-medusae Schrenk
422.	Calligonum eriopodum Bunge
423.	Calligonum junceum (Fisch. & C.A. Mey.) Litv.
424.	Calligonum leucocladum (Schrenk) Bunge
425.	Calligonum microcarpum I.G.Borshch.
426.	*Calligonum mongolicum* Turcz.
427.	*Fallopia convolvulus* (L.) A. Löve
428.	Persicaria amphibia (L.) Delarbre
429.	*Persicaria hydropiper* (L.) Delarbre
430.	*Persicaria lapathifolia* (L.) Delarbre
431.	Persicaria scabra (Moench) Moldenke
432.	*Polygonum argyrocoleon* Steud. ex Kunze
433.	Polygonum aviculare L.
434.	Polygonum patulum M.Bieb.
435.	Rheum tataricum L.fill.
436.	Rheum turkestanicum Janisch.
437.	*Rumex conglomeratus* Murray
438.	*Rumex crispus* L.
439.	*Rumex chalepensis* Mill.

440.	Rumex longifolius DC.
441.	Rumex marschallianus Reichenb.
442.	**PORTULACACEAE JUSS.**<br>Portulaca oleracea L.
443.	**POTAMOGETONACEAE**<br>*Potamogeton natans* L.
444.	*Potamogeton perfoliatus* L.
445.	*Potamogeton pusillus* L.
446.	*Stuckenia pectinata* (L.) Börner
447.	**PRIMULACEAE**<br>*Lysimachia arvensis* var. *caerulea* (L.) Turland & Bergmeier
448.	**RANUNCULACEAE JUSS.**<br>*Adonis scrobiculata* Boiss.
449.	Clematis orientalis L.
450.	*Delphinium camptocarpum* Fisch. & C.A.Mey.
451.	*Delphinium leptocarpum* (Nevski) Nevski
452.	*Delphinium rugulosum* Boiss.
453.	*Delphinium semibarbatum* Bien. ex Boiss.
454.	*Ranunculus falcatus L.*
455.	*Ranunculus learilobus* Bunge
456.	*Ranunculus repens* L.
457.	Ranunculus sceleratus L.
458.	Ranunculus testiculatis Crantz
459.	*Ranunculus trichophyllus* Chaix
460.	Thalictrum isopyroides C.A. Mey.
461.	**RHAMNACEAE JUSS.**<br>*Rhamnus erythroxyloides* subsp. *sintenisii* (Rech.f.) Mabb.
462.	Ziziphus jujuba Mill.
463.	**ROSACEAE JUSS.**<br>*Prunus spinosissima* (Bunge) Franch.
464.	Crataegus chlorocarpa Lenn, & K.Koch
465.	Crataegus pontica K.Koch
466.	*Potentilla reptans* L.
467.	Potentilla supina L.
468.	Rosa canina L.
469.	Rosa laxa Retz.
470.	Rosa majalis Herrm.
471.	*Rosa persica* Michaut ex Juss.
472.	**RUBIACEAE JUSS.**<br>*Galium aparine* L.
473.	Galium humifusum Bieb.
474.	Galium pamiroalaicum Pobed.
475.	Galium spurium L.
476.	Galium verum L.
477.	Rubia tinctorum L.
478.	**RUTACEAE JUSS.**<br>*Haplophyllum acutifolium* (DC.) G. Don
479.	Haplophyllum bungei Trautv
480.	Haplophyllum *versicolor* Fisch. & C.A. Mey.)
481.	*Haplophyllum obtusifolium* (Ledeb. ex Eichw.) Ledeb.

482.	*Haplophyllum pedicellatum* Bunge ex Boiss.
483.	Haplophyllum ramosissimum (Paulsen) Vved.
484.	Haplophyllum robustum Bunge
485.	**SALICACEAE MIRB.**<br>Populus alba L.
486.	*Populus euphratica* Olivier
487.	*Salix alba* L.
488.	Salix songarica Andersson
489.	**SCROPHULARIACEAE JUSS**.<br>*Linaria popovii* Kuprian.
490.	*Scrophularia leucoclada* Bunge
491.	*Verbascum blattaria* L.
492.	*Verbascum erianthum* Benth.
493.	*Verbascum songaricum* Schrenk
494.	Veronica campylopoda Boiss.
495.	**SOLANACEAE JUSS.**<br>Datura stramonium L.
496.	Hyoscyamus niger L.
497.	Hyoscyamus pusillus L.
498.	*Lycium dasystemum* Pojark.
499.	Lycium ruthenicum Murray
500.	Physalis praetermissa Pojark.
501.	*Solanum americanum* Mill.
502.	Solanum dulcamara L.
503.	Solanum nigrum L.
504.	**TAMARICACEAE LINK**<br>Tamarix androssowii Litv.
505.	*Tamarix aralensis* Bunge
506.	Tamarix elongata Ledeb.
507.	Tamarix hispida Willd.
508.	Tamarix hohenackeri Bunge
509.	Tamarix laxa Willd.
510.	Tamarix leptostachya Bunge
511.	Tamarix litwinowii Gorschk.
512.	Tamarix ramosissima Ledeb.
513.	**THYMELAEACEAE JUSS.**<br>Diarthron vesiculosum<br>(Fisch. & C.A.Mey.) C.A.Mey.
514.	Thymelaea passerina (L.) Coss. & Germ.
515.	**TYPHACEAE JUSS.**<br>Typha angustifolia L.
516.	Typha elephantine Roxb.
517.	Typha laxmannii Lepech.
518.	**ULMACEAE MIRB.**<br>Ulmus pumila L.
519.	Ulmus minor Mill.
520.	**URTICACEAE JUSS.**<br>Urtica dioica L.
521.	**VERBENACEAE J. ST. HILL.**<br>Verbena officinalis L.

522.	**VITACEAE JUSS**.<br>Vitis vinifera L.
523.	**ZYGOPHYLLACEAE R. BR.**<br>*Tribulus macropterus* Boiss.
524.	Tribulus terrestris L.
525.	*Zygophyllum atriplicoides* Fisch. & C.A. Mey.
526.	*Zygophyllum eichwaldii* C.A. Mey.
527.	Zygophyllum brachypterum Kar. & Kir.
528.	Zygophyllum miniatum Cham.
529.	Zygophyllum oxianum Boriss.
